# Is the Australian nursing workforce ready to embrace prescribing under supervision? A cross‐sectional survey

**DOI:** 10.1111/jan.15367

**Published:** 2022-07-19

**Authors:** Amanda Fox, Fiona Crawford‐Williams, Joseph Ria, Cardiff Lynda, Thoms Debra, Yates Patsy, Nissen Lisa, Raymond Javan Chan

**Affiliations:** ^1^ Centre for Healthcare Transformation Queensland University of Technology Brisbane Queensland Australia; ^2^ School of Nursing, Faculty of Health Queensland University of Technology Brisbane Queensland Australia; ^3^ Redcliffe Hospital Redcliffe Queensland Australia; ^4^ Caring Futures Institute, College of Nursing and Health Sciences Flinders University Adelaide South Australia Australia; ^5^ Cancer and Palliative Care Outcomes Centre, School of Nursing Queensland University of Technology Brisbane Queensland Australia; ^6^ School of Clinical Sciences, Faculty of Health Queensland University of Technology Brisbane Queensland Australia

**Keywords:** advanced practice, expanding scope, models of care, nurse, nurse prescribing, policy, workforce preparation

## Abstract

**Aim:**

The aim was to explore nurses' preparedness to expand their practice to prescribe medicines under a supervision model.

**Design:**

This was a cross‐sectional study.

**Methods:**

A convenience sample of Australian nurses recruited from memberships of State‐based Nursing and Midwifery Unions and professional bodies from diverse care settings. Nurses undertook an online researcher‐constructed survey between March and July 2021 to identify current prescribing practices, motivations for undertaking education in prescribing and perceived barriers to implementation of nurse prescribing under supervision. Data related to demographics, nursing experience and barriers to becoming a prescriber were analysed descriptively. Logistic regression was used to model nursing experience variables with desire to become a prescriber.

**Results:**

A total of 4424 nurses participated with the majority (*n* = 3645, 82%) reporting they were highly likely to expand their practice to prescribe medicines under supervision. The main motivations to prescribe were to enhance patient care and job satisfaction. Nurses were more likely to want to prescribe if they had <10 years experience (95% CI = 0.3–0.5, *p* < 0.001), held a bachelor's degree (95% CI = 1.3–2.2, *p* < 0.001) or higher qualification (95% CI = 1.8–2.9, *p* < 0.001). Most reported lack of acknowledgement of increased responsibility and workloads (*n* = 4098, 93%), and insufficient organizational support (*n* = 4197, 95%) may prevent uptake of nurse prescribing.

**Conclusions:**

Most Australian nurses demonstrated their preparedness to embrace the role of prescribing under supervision. The perceived barriers identified in this study can inform future implementation of this expanded nursing role.

**Impact:**

The Nursing and Midwifery Board of Australia has proposed a standard of practice to enable nurses to prescribe under supervision. Models of nurse prescribing are being considered globally to address population needs. Successful adoption of this practice is dependent on aspects such as key personnel's acceptance of the initiative. The workforce readiness and barriers highlighted in this study can inform implementation at policy and organizational levels.

## 1 INTRODUCTION

Extension of nursing roles has been touted as the quickest and most cost‐effective means to universal health coverage (Crisp et al., 2018). Universal health coverage ensures that individuals are provided with quality health services based on their health needs without financial hardship (WHO, 2021). The International Council of Nurses (ICN) has made a global call to action and published guidelines on prescriptive authority for nurses to facilitate a common understanding of nurse prescribing to enhance access to medicine, improve patient outcomes and optimize the knowledge and skills of the nursing workforce (Stewart et al., 2021). The ICN recognize that although labelled with various titles, nurse prescribing practice falls into three categories, independent, supplementary and prescribing via a protocol or arrangement (Stewart et al., 2021). Nurse prescribing has been implemented in many countries including Canada, China, Cyprus, Denmark, Finland, Israel, New Zealand, Sweden, United Kingdom and others (Ladd & Schober, 2018). Role expansion is occurring worldwide (Maier, 2019); however, reframing professional boundaries requires associated changes to legislation and policy (Niezen & Mathijssen, 2014). In 2018, the Nursing and Midwifery Board of Australia proposed the introduction of a Registered Nurse Prescribing Standard of Practice according to a supervised model (NMBA, 2018). Within this model, independent prescribers are practitioners with the authority to prescribe medicines autonomously (i.e. medical practitioners, nurse practitioners). The supervised model reflects the Health Professionals Prescribing Pathway Model 2 and will enable registered nurses to prescribe under the supervision of an independent prescriber (HWA, 2013).

## 2 BACKGROUND

Globally, nurses practise within varying prescribing models. In some countries such as Australia, Canada, New Zealand and the United States of America, a limited number of master's degree qualified, advanced practice nurses are authorized to independently prescribe medicine (Maier & Aiken, 2016). Other countries employ a supported model involving the nurse and an independent prescriber, for example, the task sharing model adopted in Madagascar, Ethiopia and Pakistan (Ladd & Schober, 2018). Non‐medical professionals have been prescribing according to both independent and supplementary prescribing models for over 30 years in the United Kingdom, (Maier, 2019) where this practice is well accepted by clinicians and patients and provides benefits for patients, the organization and nurses (Armstrong, 2015; Casey et al., 2019; Gielen et al., 2014). Role expansion to include prescribing in most cases is preceded by the need for nurses to complete specific prescriber education.

Despite the success of nurse prescribing in the United Kingdom, some healthcare settings have experienced limited, or slow uptake of non‐medical prescribing associated with nurse concerns about inadequate educational preparation and remuneration for the additional responsibilities (Boreham et al., 2013; Ross & Kettles, 2012). Lennon and Fallon (2018) report that around 57% of Registered Nurse Prescribers in Ireland were using their prescriptive authority. Studies conducted in Ireland report that motivation to undertake prescribing educational programs and accept increased levels of responsibility is driven by the desire to improve patient outcomes and job satisfaction (Casey et al., 2019; Lennon & Fallon, 2018). Alternatively, there are concerns about increased workload and stress associated with prescribing medicine (Armstrong, 2015; Maddox et al., 2016).

Whilst nurse practitioners in Australia are authorized to prescribe independently, registered nurses do not currently have prescribing rights. In a study of Australian mental healthcare professionals (49 nurses, 7 medical practitioners, 26 allied health professionals), participants reported that the introduction of nurse prescribing under supervision would potentially improve patient access to medicines but also voiced concerns about prescribing accuracy and patient safety (Muyambi et al., 2018). Research on nurse prescribing is dominated by the United Kingdom experience and whilst this is valuable, exploring local contextual considerations and involving key personnel during implementation is imperative (Fox et al., 2021).

## 3 THE STUDY

### 3.1 Aim

The aim of this research was to provide a national overview of registered nurses' views towards implementation of nurse prescribing in Australia. Specifically, the study aimed to identify nurses' acceptance of the prescribing under supervision model, current prescribing practices, predictors of willingness to prescribe, motivation to undertake education in prescribing and perceived barriers to implementation of nurse prescribing under supervision.

### 3.2 Design

A national survey was conducted between March and July 2021 and is reported here according to the STROBE statement for cross‐sectional studies where relevant (von Elm, Altman, Egger, et al., 2007). This study mainly used a quantitative approach with sections where further free‐text responses were permitted and encouraged.

### 3.3 Participants

A convenience sample of nurses registered with the Nursing and Midwifery Board of Australia were invited to take part in this research. Enrolled nurses (diploma‐prepared nurses who practise within their scope of practice under the direct/indirect supervision of a registered nurse), registered midwives who were not registered nurses, student nurses and assistants in nursing were excluded. As the primary aim of the study was exploratory and descriptive, no ‘a priori’ sample size was calculated; however, a final sample with a minimum of 500 participants was sufficient to adequately power the logistic regression analyses (Bujang et al., 2018).

### 3.4 Data collection, validity and reliability

An online survey was developed by the research team (RJC, AF, RJ, LC, PY, DT, LN) comprising senior academics with expertise in nursing, nurse‐led models of care, policy, pharmacy and non‐medical prescribing and informed by a recent integrative review conducted by the research team (Fox et al., 2021). The content validity of the initial survey was assessed by 10 experts who participated as panel members using the Content Validity Index in terms of relevance and clarity (Grant & Davis, 1997; Lynn, 1986). Criteria for selection of the panel included having: (i) a minimum education level of a master's degree, (ii) a minimum of 3 years of experience in nursing/pharmacy/implementation science/survey design. Informed by the qualitative comments of the Expert Panel, changes were made to the survey by the research team where there was no consensus on the relevance and clarity of items. Subsequently, the updated survey was distributed to 10 registered nurses who checked usability and face validity (time needed to complete the questionnaire; their views on clarity of questions and whether the questions are understandable and easy to answer). With the feedback from these 10 nurses, further minor changes were made to the survey to enhance clarity.

The final 32‐item survey comprised three sections, Section A: demographic characteristics and nursing experience, Section B: views towards nurse prescribing as a role expansion and Section C: beliefs around educational requirements for the extended role including qualifications and continuing professional development. The results presented in this manuscript relate to Section B which included six questions. Participants’ views about the expected outcomes of nurse prescribing under supervision (11 items) and factors enabling implementation of nurse prescribing (12 items) were measured on a five‐point Likert response scale with responses 0 = *strongly disagree*, *1 = disagree*, *2 = neutral or disagree*, *3 = agree* to 4 = *strongly agree* to statements such as *Implementing nurse prescribing will improve healthcare delivery* and *Support from colleagues in the nursing profession will enable nurse prescribing under supervision*. The likelihood of wanting to become a prescriber if nurses were able to complete education was measured in a single‐item question with five responses: 1 = *extremely unlikely*, *2 = somewhat unlikely*, *3 = neither likely nor unlikely*, *4 = somewhat likely*, 5 = *extremely likely*. Current prescribing practices were grouped into five categories based on the Health Professionals Prescribing Pathway Project (HWA, 2013) definition and included: independent prescribing, initiating medicine based on a protocol or formulary, adjusting medicine based on a protocol or formulary, ceasing patient medicines or not prescribing medicine with participants able to select multiple options. Participants were provided with five motivations for becoming a prescriber and asked to rank the items from 1 = *highest motivation* to 5 = *lowest motivation*. Finally, participants were asked to select from a list of seven items, those that would make them unlikely to want to become a prescriber, with participants able to select multiple.

The final survey was uploaded into Key Survey. An invitation email containing the survey link was distributed to the members of four state‐based nursing and midwifery unions including the Queensland Nurses and Midwives' Union, the New South Wales Nurses and Midwives' Association, the Australian Nursing and Midwifery Federation ‐Victoria and the Australian Nursing and Midwifery Federation ‐South Australia, professional nursing bodies and networks of the research team. The survey was anonymous and no identifiable information was collected from participants, ensuring participant confidentiality.

### 3.5 Ethical considerations

Ethics approval was granted by the Queensland University of Technology Human Research Ethics Committee (#2000000418).

### 3.6 Data analysis

All statistical analyses were performed using IBM SPSS Statistics (version 27). Descriptive statistics were calculated (count and percentage) for demographic data, data relating to nursing experience and items that made it unlikely for nurses to want to become a prescriber. Current prescribing practices across states, qualification level and years of experience were explored by computing cross‐tabulations. Counts and percentages as well as means and standard deviations were calculated for Likert scale items relating to participant views and ranked motivations. Normality of the distribution was tested with the absolute values of the skewness and kurtosis because of the large sample size (Kim, 2013). All data were normally distributed.

A binary variable was created to represent whether nurses wanted to become a prescriber or not, with responses to the item ‘If nurses were able to complete education to prescribe medicines, how likely are you to want to become a prescriber?’ coded as ‘*No desire’* = extremely unlikely, unlikely and neutral and ‘*desire’* = extremely likely and likely. Univariate and multivariate logistic regression was used to model nursing experience variables (level of qualification, years of experience, workplace setting) and state with the desire to become a prescriber. These variables were included as past research has indicated that experience has an impact on views towards nurse prescribing (Ling et al., 2021). Statistical significance was defined as *p* < 0.05. Missing data were deemed ‘missing completely at random’, and as such, only complete case analysis was carried out.

## 4 RESULTS

### 4.1 Demographic characteristics

A total of 4424 registered nurses participated in the study, representing approximately 1.3% of all registered nurses in Australia (AGDoH, 2019). Most participants were from New South Wales (*n* = 3009, 69.2%), over 40 years of age (*n* = 2828, 63.92%), possessed a bachelor's degree or higher (*n* = 3594, 89.38%) and received their nursing qualification in Australia (*n* = 3702, 83.67%). Half of the participants were employed full‐time (*n* = 2213, 50.02%), and participants worked predominantly in public hospitals (*n* = 2744, 62.02%), in inpatient settings (*n* = 2416, 73.14%) and in clinical roles (*n* = 3711, 83.88%). Participants were employed in a wide variety of practice contexts, with a large proportion working in medical, surgical, community, primary health and emergency areas. Table 1 provides detailed information about the demographic characteristics and nursing roles of the nurse participants.

**TABLE 1:** Demographic and workforce characteristics of the sample

Age	*N*	%
20–29	516	11.7%
30–39	1054	23.8%
40–49	942	21.3%
50–59	1162	26.3%
>60	724	16.4%
Aboriginal or Torres Strait Islander		
Yes	106	2.4%
No	4426	95.6%
Prefer not to answer	87	2.0%
State		
NSW	3009	69.2%
QLD	708	16.3%
VIC	291	6.7%
WA	194	4.5%
SA	108	2.5%
Other (TAS, NT, ACT)	36	0.8%
Country of qualification		
Australia	3702	84.1%
Overseas	701	15.9%
Highest qualification		
Hospital certificate or diploma	467	10.5%
Bachelor's degree	1606	36.3%
Post‐graduate certificate or higher	2348	53.1%
Years of experience		
<5 years	677	15.3%
5 to <10 years	693	15.6%
10 to <15 years	643	14.5%
15 to <20 years	485	10.9%
20 or more years	1919	43.4%
Context of practice		
Acute care	1247	28.1%
Community or primary health	637	14.4%
Emergency	484	10.9%
Gerontology/aged care	376	8.5%
Mental health/alcohol and other drugs	373	8.4%
Women and children's health	329	7.4%
Cancer and palliative care	264	5.9%
Chronic health care	254	5.7%
Education, administration or management	128	2.9%
Other	326	7.4%
Workplace setting		
Public hospital	2744	62.0%
Private hospital	438	9.9%
Community or primary health service	544	12.3%
Residential aged care or retirement village	349	7.9%
Other	349	7.9%
Patient group^a^ (*N* = 4021)		
Adults	2895	71.9%
Across the lifespan	752	18.7%
Paediatrics	346	8.6%
Neonatal	207	5.1%
Nursing role^a^ (*N* = 4424)		
Clinical	3711	83.9%
Administrative/managerial	702	15.9%
Academic/education	602	13.6%
Research	106	2.4%
Other	103	2.3%

^a^ Participants could select more than one thus % does not equal 100.

### 4.2 Prescribing practices

Current prescribing practices are reported in Table 2. The majority of nurses were not prescribing medicines (*n* = 3163, 71.49%,) with just over one‐quarter of nurses involved in initiating medicines based on a protocol or formulary (*n* = 1166, 26.35%). Prescribing practices appeared to vary according to state, years of experience and level of qualification with larger proportions of nurses prescribing, initiating, adjusting or ceasing medication in Queensland and New South Wales, amongst those with a post‐graduate qualification or higher, and for participants with less than 10 years nursing experience.

**TABLE 2:** Current prescribing practices of sample across state, qualification level and years of experience

Do you currently prescribe?^a^	Total sample	NSW	QLD	VIC	WA	SA, ACT NT, TAS	Cert or diploma	Bachelor degree	Post‐grad or higher	<10 years exp	>10 years exp
No. of participants	4424	3009	708	291	194	144	467	1606	2348	1370	3047
Yes I prescribe independently	58 (1.3%)	41 (1.4%)	11 (1.5%)	3 (1.0%)	2 (1.0%)	0	2 (0.4%)	25 (1.6%)	31 (1.3%)	23 (2.2%)	35 (1.1%)
No but I initiate medicine based on a protocol or formulary	1166 (26.3%)	807 (26.8%)	202 (28.5%)	65 (22.3%)	49 (25.2%)	35 (24.3%)	110 (23.5%)	414 (25.8%)	642 (27.3%)	420 (30.7%)	745 (24.4%)
No but I adjust based on a protocol or formulary	289 (6.5%)	215 (7.1%)	31 (4.4%)	21 (7.2%)	10 (5.2%)	9 (6.2%)	18 (3.9%)	67 (4.2%)	204 (8.7%)	101 (7.3%)	188 (6.2%)
No but I cease patient medicines	82 (1.9%)	56 (1.9%)	14 (2.0%)	8 (2.7%)	1 (0.5%)	2 (1.4%)	4 (0.9%)	22 (1.4%)	56 (2.4%)	32 (2.3%)	50 (1.6%)
No I do not prescribe	3163 (71.5%)	2134 (70.9%)	493 (69.6%)	217 (74.5%)	144 (74.2%)	106 (73.6%)	352 (75.4%)	1177 (73.4%)	1631 (69.5%)	936 (68.3%)	2221 (72.9%)

^a^ Participants could select more than one thus % does not add up to10.

The shaded row indicates the initial question in the survey and how responses were compared across state, education and experience.

### 4.3 Views about implementation of nurse prescribing

Participants’ views about the implementation of nurse prescribing under supervision and the factors that may enable this practice are shown in Table 3. In general, participants felt that implementing nurse prescribing would *improve the use of nurses' knowledge*, *skills and capability* (92% agree or strongly agree), *increase access to nurse‐led models of care* (90% agree or strongly agree) and *improve patients' healthcare experience* (86% agree or strongly agree). The highest rated factors that would enable implementation of nurse prescribing included *supportive legislation and policy* (92% agree or strongly agree), *appropriate mentors* (91% agree or strongly agree) and *models of nursing care that optimize use of nurse prescribing* (92% agree or strongly agree).

**TABLE 3:** Participants' attitudes towards implementation and enablers of registered nurse prescribing ^╪^ scale ranked 0–4

	Mean (*SD*)^╪^	Strongly disagree	Disagree	Neutral	Agree	Strongly agree
Implementing registered nurse prescribing will:						
Improve use of registered nurse knowledge, skills and capability	3.46 (0.75)	39 (0.9%)	64 (1.5%)	245 (5.6%)	1529 (34.9%)	2501 (57.1%)
2 Increase access to nurse‐led models of care	3.40 (0.77)	42 (1.0%)	76 (1.7%)	294 (6.7%)	1641 (37.4%)	2330 (52.7%)
3 Improve patient healthcare experience	3.31 (0.84)	51 (1.2%)	114 (2.6%)	442 (10.1%)	1588 (36.3%)	2183 (49.9%)
4 Improve capacity of the Australian healthcare system due to a more flexible workforce	3.27 (0.89)	64 (1.5%)	138 (3.2%)	490 (11.2%)	1536 (35.2%)	2138 (49.0%)
5 Improve healthcare delivery	3.26 (0.86)	56 (1.3%)	138 (3.1%)	456 (10.4%)	1691 (38.4%)	2058 (46.8%)
6 Improve patient education regarding medicines	3.25 (0.88)	53 (1.2%)	143 (3.3%)	528 (12.1%)	1574 (35.9%)	2081 (47.5%)
7 Improve patient access to prescription medicines	3.18 (0.90)	48 (1.1%)	171 (3.9%)	645 (14.7%)	1608 (36.7%)	1914 (43.6%)
8 Improve retention of clinicians within the nursing profession	3.04 (1.00)	71 (1.6%)	249 (5.7%)	949 (21.7%)	1280 (29.3%)	1824 (41.7%)
9 Reduce costs to the Australian healthcare system	3.03 (0.98)	66 (1.5%)	205 (4.7%)	1029 (23.6%)	1300 (29.9%)	1753 (40.3%)
10 Reduce healthcare costs to the patient	2.90 (1.01)	57 (1.3%)	280 (6.4%)	1297 (29.6%)	1175 (26.8%)	1574 (35.9%)
11 Reduce safety risks for patients	2.61 (1.07)	126 (2.9%)	450 (10.3%)	1440 (33.0%)	1231 (28.2%)	1118 (25.6%)
Factors enabling registered nurse prescribing are:						
Supportive legislation, regulation and relevant health policy	3.54 (0.74)	39 (0.9%)	46 (1.1%)	274 (6.3%)	1172 (27.0%)	2812 (64.7%)
2 Availability of appropriate mentors and/or supervisors to facilitate role, skill and knowledge development	3.49 (0.77)	44 (1.0%)	77 (1.8%)	259 (5.9%)	1296 (29.6%)	2696 (61.7%)
3 Models of nursing care that optimize use of nurse prescribing	3.49 (0.74)	40 (0.9%)	42 (1.0%)	282 (6.5%)	1378 (31.7%)	2605 (59.9%)
4 Acknowledgement of the impact on workload of registered nurse	3.44 (0.84)	63 (1.4%)	111 (2.5%)	297 (6.8%)	1296 (29.6%)	2616 (59.7%)
5 Support from pharmacy colleagues	3.44 (0.84)	58 (1.3%)	97 (2.2%)	346 (7.9%)	1237 (28.3%)	2631 (60.2%)
6 Support from medical colleagues	3.43 (0.88)	69 (1.6%)	150 (3.4%)	312 (7.1%)	1159 (26.4%)	2693 (61.4%)
7 Organizational commitment for implementation	3.42 (0.78)	41 (0.9%)	66 (1.5%)	354 (8.1%)	1459 (33.0%)	2463 (56.2%)
8 Support from colleagues in the nursing profession	3.40 (0.75)	38 (0.9%)	54 (1.2%)	317 (7.3%)	1687 (38.6%)	2272 (52.0%)
9 Acceptance of nurse prescribing by patients/clients	3.32 (0.82)	53 (1.2%)	74 (1.7%)	449 (10.3%)	1607 (36.9%)	2171 (49.9%)
10 Remuneration to acknowledge prescribing practice	3.28 (0.88)	60 (1.4%)	103 (2.4%)	584 (13.4%)	1404 (32.2%)	2213 (50.7%)
11 Health services receive reimbursement for registered nurse prescribing activities	3.11 (0.90)	51 (1.2%)	127 (2.9%)	860 (19.7%)	1569 (36.0%)	1757 (40.3%)
12 Unrestricted prescribing based on a clear scope of practice	2.87 (1.05)	101 (2.3%)	382 (8.8%)	966 (22.2%)	1423 (32.6%)	1487 (34.1%)

A majority of participants (*n* = 3309, 80.84%) felt that patients who receive a prescription from a nurse prescriber (where a fee is charged to the patient), should be eligible for the Medicare Benefits Schedule, and a similar number (*n* = 3349, 82.06%) felt that patient access to the Pharmaceutical Benefits Scheme should be facilitated by the inclusion of nurse prescribers for relevant medicines. *Note: The provision of healthcare in Australia is subsidized by the government. The Medicare Benefits Schedule and Pharmaceutical Benefits Scheme detail the medical services and medicines eligible for subsidy*, *respectively*.

A high proportion of respondents indicated that they would be likely to become a prescriber if specific education was available (82% somewhat or extremely likely), although proportions varied across age groups, states and qualification level. At the univariate level, wanting to become a prescriber was significantly associated with state, level of qualification, years of experience and workplace setting. Adjusted odds ratios (Table 4) indicated that participants were statistically significantly more likely to report wanting to become a prescriber if they lived in Victoria, (OR = 2.1, 95% CI = 1.3–3.2, *p* < 0.001) and had higher education qualifications (Bachelor's degree: OR = 1.7, 95% CI = 1.3–2.2, *p* < 0.001 and post‐graduate or higher: OR = 2.3, 95% CI = 1.8–2.9, *p* < 0.001). Participants with more than 10 years of nursing experience were statistically significantly less likely to report wanting to become a prescriber compared with those with less than 10 years experience (OR = 0.4; 95% CI = 0.3–0.5, *p* < 0.001), and if they worked in settings outside of public, private, community or aged care (OR = 0.7, 95% CI = 0.5–0.9, *p* = 0.009).

**TABLE 4:** Crude and adjusted odds ratios from logistic regression analyses identifying associations between selected nursing characteristics and desire to become a prescriber, *n* = 4334

Variable	No. of participants	Proportion wanting to prescribe	Crude odds ratio	Adjusted odds ratio	95% CI	*p*
State						
NSW	3001	81.9%	1.0	1.0		
Victoria	291	91.1%	2.2	2.1	1.3–3.2	<0.001
Queensland	705	80.6%	0.9	0.8	0.7–1.1	0.139
Western Australia	193	82.7%	1.6	1.5	0.9–2.4	0.565
Other (SA, TAS, NT, ACT)	144	83.3%	1.1	1.1	0.7–1.8	0.061
Qualification						
Certificate or Diploma	448	67.0%	1.0	1.0		
Bachelor's degree	1580	84.2%	2.6	1.7	1.3–2.2	<0.001
Post‐graduate or higher	2306	82.5%	2.7	2.3	1.8–2.9	<0.001
Years of experience						
0 to <10 years	1356	90.9%	1.0	1.0		
10 or more years	2978	78.6%	0.4	0.4	0.3–0.5	<0.001
Workplace setting						
Public hospitals	2701	83.2%	1.0	1.0		
Private hospitals	433	80.5%	0.8	0.8	0.6–1.1	0.230
Community or primary care	532	84.7%	1.1	1.3	0.9–1.6	0.080
Aged care	336	83.1%	0.9	1.2	0.8–1.6	0.386
Other	332	74.4%	0.6	0.7	0.5–0.9	0.009

For participants who indicated they would want to become a prescriber if education was available, the highest ranked motivation was for *improved care to patients* (*M* = 3.44, *SD* = 1.09), followed by *improved job satisfaction* (*M* = 2.28, *SD* = 1.10), *improved professional reputation* (*M* = 1.66, *SD* = 1.14) and *contribution to the multi‐disciplinary team* (*M* = 1.61, *SD* = 1.29). *Increased remuneration* was the lowest motivator in relative terms (*M* = 1.10, *SD* = 1.22).

The most common reasons for participants to be unlikely to become a prescriber are available in Supplementary Table S1 and included: *not believing there would be organizational support* (43.15%), *not changing the patient care they provide* (21.02%) and *not being prepared to take on extra responsibility* (15.80%).

## 5 DISCUSSION

To our knowledge, this is the first study in Australia to explore the readiness of the nursing workforce to embrace prescribing under supervision. We believe the findings from this study are *not only* relevant to the nursing profession, but to a range of stakeholders including policymakers; independent prescribers (i.e. medical and nurse practitioners) and healthcare organizations planning to introduce expanded prescribing practices. The vast majority of the participants expressed the high likelihood of undertaking further study to expand their practice to prescribe medicine under a supervision model. Most nurses working in Australia do not currently prescribe medicines, however as many as one‐third are already engaged in initiating, adjusting or ceasing medicines using formulary and protocols.

Compared to other states, nurses working in Victoria were most likely to have the desire to become prescribers. It is difficult to speculate the true reason. It is important that future research explores system‐level contextual factors. Participants aged less than 50 years, with less than 10 years experience and those with higher qualifications were more likely to want to become a prescriber than nurses over the age of 50 years, with more than 10 years experience or those with certificate level qualifications. This finding is consistent with research by Pool et al. (2015) who found that age influences a nurse's motivation to undertake ongoing education, in particular that younger nurses were motivated to gain experience and build on their career. Additionally, participants expressed strongly the need for changes to funding schedules such as patient access to the Medicare Benefits Schedule and the Pharmaceutical Benefits Scheme. This will require significant legislative review and collaboration to ensure a workable solution.

Nurses perceived that prescribing under supervision would improve use of nurse knowledge, skills and capability, as well as support nurse‐led models of care improving patient experiences. Their key motivation to prescribe were improving patient care and gaining greater job satisfaction. Those who were unlikely to become a prescriber did not believe their organization would support it. Evidence suggests that lack of organizational support for service innovation will prevent initial adoption and sustainability (Fox et al., 2017), suggesting that successful implementation will require guidance for organizational support.

The results of this national study are consistent with research undertaken in the United Kingdom where nurses reported greater work satisfaction and being motivated by the opportunity to improve access to medicine for patients (Casey et al., 2019; Gielen et al., 2014). However, this research highlights the desire of nurses to expand their practice but also the need for support from colleagues. Research undertaken post implementation of independent nurse prescribing and broader non‐medical prescribing models in the United Kingdom has shown that financial recognition of the increased responsibility is desired (Maddox et al., 2016). An Australian study exploring the perspectives of 1205 registered nurses on expanding their scope of practice (not specific to prescribing) reported the most frequently cited barrier was a lack of financial incentive, organizational guidelines and support (Muyambi et al., 2018).

As the NMBA progresses, the relevant standard in this study indicates support from the profession for the planned change. The implementation of nurse prescribing under supervision is also dependent upon independent prescribers' preparedness to supervise nurses' training and practice and internal organizational structures to support this practice. To date, no study has been published that explores the motivations or willingness of independent prescribers or healthcare organizations to support the introduction of supervised nurse prescribing. Along with supportive policy and legislation, understanding the view of all key stakeholders prior to implementation will enable successful implementation of this nurse expanded scope of practice. It is hypothesized that better planning will facilitate better adoption of prescribing under supervision.

### 5.1 Limitations

The results of this survey are limited to the participants who took part and therefore participant bias of those most interested in prescribing may have impacted the findings of this study. Small participant numbers in some states may not have been representative of the broader population of nurses. Therefore, it may not necessarily be fully generalizable. There are some limitations based on the cross‐sectional nature of the study, as it is difficult to interpret the cause of the identified associations. Further, there are limitations to the interpretation of the data for some questions, for instance, the ranking of motivations for becoming a nurse prescriber were not completed by the whole sample, and the ranking process does not allow for an objective indicator of how participants felt about each item.

## 6 CONCLUSION

This study is the first to explore the attitude of Australian registered nurses towards nurse prescribing under supervision and their desire to become a prescriber. As such, it provides necessary baseline information to inform the progress of nurse prescribing in Australia. Findings from this study clearly indicate a willingness of the majority of nurses to expand their practice to include prescribing. The enablers and barriers reported in this study have implications for future implementation initiatives, research and policy development and highlight the need for an effective legislative framework and regulatory endorsement, appropriate education, training and mentorship, supportive organizational policies and reforms to nursing models of care that facilitate nurse prescribing.

## AUTHOR CONTRIBUTIONS

AF, RC, FCW: Made substantial contributions to conception and design, or acquisition of data, or analysis and interpretation of data; AF, RC, FCW: Involved in drafting the manuscript or revising it critically for important intellectual content; AF, FCW, RJ, LC, DT, PY, LN & RC: Given final approval of the version to be published. Each author should have participated sufficiently in the work to take public responsibility for appropriate portions of the content; AF, FCW, RJ, LC, DT, PY, LN & RC: Agreed to be accountable for all aspects of the work in ensuring that questions related to the accuracy or integrity of any part of the work are appropriately investigated and resolved.

## FUNDING INFORMATION

RJC receives salary support from the National Health and Medical Research Council (APP1194051).

## CONFLICT OF INTEREST

None.

### PEER REVIEW

The peer review history for this article is available at https://publons.com/publon/10.1111/jan.15367.

## Supporting information


Table S1

## Data Availability

Data used and collected whilst conducting this research are available on request. Please contact the corresponding author for more details.
