# Supplementary material for: Is the Australian nursing workforce ready to embrace prescribing under supervision? A cross‐sectional survey
Source: J Adv Nurs. 2022 Jul 19;78(12):4082–91. doi: 10.1111/jan.15367 (PMC9796876; doi:10.1111/jan.15367)
Supplement: Supplementary file 1 — Table S1 [file JAN-78-4082-s001.docx]

**Table S1.** Likelihood of wanting to become a prescriber according to demographic and workplace characteristics

|  | **No. of participants** | **Extremely unlikely** | **Somewhat unlikely** | **Neither likely or unlikely** | **Somewhat likely** | **Extremely likely** |
| --- | --- | --- | --- | --- | --- | --- |
| **Total sample** | 4421 | 245 (5.5%) | 189 (4.3%) | 342 (7.7%) | 1142 (25.8%) | 2503 (56.6%) |
| **Age** |  |  |  |  |  |  |
| 20 – 29 | 515 | 11 (2.1%) | 13 (2.5%) | 21 (4.1%) | 123 (23.9%) | 347 (67.4%) |
| 30-39 | 1054 | 25 (2.4%) | 38 (3.6%) | 42 (3.9%) | 248 (23.5%) | 701 (66.5%) |
| 40-49 | 942 | 32 (3.4%) | 27 (2.9%) | 69 (7.3%) | 236 (25.1%) | 578 (61.3%) |
| 50-59 | 1160 | 89 (7.7%) | 63 (5.4%) | 108 (9.3%) | 322 (27.8%) | 578 (49.8%) |
| 60 or over | 724 | 86 (11.9%) | 47 (6.5%) | 97 (13.4%) | 204 (28.2%) | 290 (40.1%) |
| **State** |  |  |  |  |  |  |
| NSW | 3007 | 178 (5.9%) | 128 (4.3%) | 237 (7.9%) | 774 (25.7%) | 1690 (56.2%) |
| QLD | 707 | 35 (5.0%) | 44 (6.2%) | 58 (8.2%) | 177 (25.0%) | 393 (55.6%) |
| VIC | 291 | 7 (2.4%) | 6 (2.1%) | 13 (4.5%) | 66 (22.7%) | 199 (68.4%) |
| WA | 194 | 4 (2.1%) | 7 (3.6%) | 12 (6.2%) | 64 (33.0%) | 107 (55.2%) |
| SA, ACT, NT, TAS | 144 | 9 (6.3%) | 2 (1.4%) | 13 (9.0%) | 37 (25.7%) | 83 (57.6%) |
| **Qualification** |  |  |  |  |  |  |
| Certificate or Diploma | 466 | 56 (12.0%) | 41 (8.8%) | 57 (12.2%) | 146 (31.3%) | 166 (35.6%) |
| Bachelor’s degree | 1606 | 67 (4.2%) | 63 (3.9%) | 124 (7.7%) | 431 (26.8%) | 921 (57.3%) |
| Post-grad or higher | 2346 | 121 (5.2%) | 84 (3.6%) | 161 (6.9%) | 564 (24.0%) | 1416 (60.4%) |
| **Country of qualification** |  |  |  |  |  |  |
| Australia | 3700 | 214 (5.8%) | 154 (4.2%) | 284 (7.7%) | 953 (25.8%) | 2095 (56.6%) |
| Overseas | 700 | 30 (4.3%) | 35 (5.0%) | 56 (8.0%) | 185 (26.4%) | 394 (56.3%) |
| **Years of experience** |  |  |  |  |  |  |
| <5 | 677 | 12 (1.8%) | 20 (3.0%) | 36 (5.3%) | 160 (23.6%) | 449 (66.3%) |
| 5 to <10 | 692 | 18 (2.6%) | 16 (2.3%) | 22 (3.2%) | 163 (23.6%) | 473 (68.4%) |
| 10 to <15 | 643 | 28 (4.4%) | 24 (3.7%) | 44 (6.8%) | 152 (23.6%) | 395 (61.4%) |
| 15 to <20 | 485 | 24 (4.9%) | 22 (4.5%) | 38 (7.8%) | 130 (26.8%) | 271 (55.9%) |
| 20 or more | 1917 | 163 (8.5%) | 107 (5.6%) | 201 (10.5%) | 534 (27.9%) | 912 (47.6%) |
| **Workplace setting** |  |  |  |  |  |  |
| Public hospital | 2743 | 135 (4.9%) | 111 (4.0%) | 214 (7.8%) | 738 (26.9%) | 1545 (56.3%) |
| Private hospital | 437 | 32 (7.3%) | 22 (5.0%) | 31 (7.1%) | 110 (25.2%) | 242 (55.4%) |
| Community or primary health | 544 | 22 (4.0%) | 25 (4.6%) | 36 (6.6%) | 137 (25.2%) | 324 (59.6%) |
| Residential aged care | 349 | 18 (5.2%) | 17 (4.9%) | 24 (6.9%) | 78 (22.3%) | 212 (60.7%) |
| Other | 348 | 38 (10.9%) | 14 (4.0%) | 37 (10.6%) | 79 (22.7%) | 180 (51.7%) |
